# Supplementary material for: Assessment of Infection Prevention and Control Measures at Points of Entry in Sierra Leone in 2021: A Cross-Sectional Study
Source: Int J Environ Res Public Health. 2022 May 13;19(10):5936. doi: 10.3390/ijerph19105936 (PMC9140457; doi:10.3390/ijerph19105936)
Supplement: Supplementary file 1 [file ijerph-19-05936-s001.zip › ijerph-1635641-supplementary.pdf]

Supplementary Materials

Table S1: Summary of the study sites

Region	District	PoE Type	PoE Name	Number of PoE	PoE Class
Western	Western Area Urban	Seaport	Queen Elizabeth II Quay	1	Class A
Northwest	Port Loko	Airport	Lungi International	1	Class A
	Kambia	Ground crossing	Gbalamuya	1	Class A
Southern	Pujehun	Ground crossing	Jendema	1	Class A

Table S2:

A CHECKLIST ADAPTED FROM THE WHO INFECTION PREVENTION AND CONTROL TOOL USED TO ASSESS IPC STATUS AT THE FOUR CLASS A POINTS OF ENTRIES IN SIERRA LEONE

Purpose: Assessment of infection prevention and control Measures at Points of Entry (PoE) in Sierra Leone in 2021: a cross-sectional study

1. General Information:

Date [/ /]   District [ ]   City/Town/Village [ ]

PoE name [ ]   PoE type [ ]   PoE class [ ]

Name & Designation of interviewer [ ]   Contact number [ ]   E-mail address [ ]

Name & Designation of respondent [ ]   Contact number [ ]   E-mail address [ ]

2. Infection Prevention and Control (IPC) Components

i. IPC guidelines

PoEs should have guidelines on IPC that align with and contribute towards the prevention of infections and antimicrobial resistance in the environment

No.	Questions	Answer	Score
1	Does the PoE have guidelines on the following? *(Indicate **YES** only if guideline is available)*		
i	Screening of travelers upon arrival or departure	☐ Yes	1
		☐ No	0
ii	Isolation of suspected/sick travelers	☐ Yes	1
		☐ No	0
iii	Referral of suspected/sick travelers	☐ Yes	1
		☐ No	0
iv	Hand hygiene	☐ Yes	1
		☐ No	0
v	Outbreak management and preparedness	☐ Yes	1
		☐ No	0
vi	Cleaning and disinfection	☐ Yes	1
		☐ No	0
vii	Port health staff protection and safety	☐ Yes	1
		☐ No	0
viii	Waste management	☐ Yes	1
		☐ No	0
2	If yes, are the guidelines consistent with national/international guidelines	☐ Yes	1
		☐ No	0
3	Are port health staff involved in both planning and executing the implementation of IPC guidelines in addition to IPC personnel?	☐ Yes	1
		☐ No	0
4	Are relevant stakeholders (for example, clinicians, EPR personnel, port managers) involved in the development and adaptation of the IPC guidelines in addition to IPC personnel?	☐ Yes	1
		☐ No	0
5	Do port staff receive specific training related to new or updated IPC guidelines introduced at the PoE?	☐ Yes	1
		☐ No	0
	**Subtotal score**	**/12**	

ii. IPC training

PoE staff should be trained on general principles of IPC

No.	Questions	Answer	Score
1	Are there port health staff trained on basic IPC? (If yes state when)	☐ Yes	1
		☐ No	0
2	If yes, how frequently do port health staff receive training regarding IPC in your station?	☐ Never or rarely	0
		☐ New employee orientation	0.2
		☐ New employee orientation and regular (at least annually) IPC training but not mandatory	0.5
		☐ New employee orientation and regular (at least annually) mandatory IPC training	1
3	If yes, are port health staff trained through interactive training sessions (for example, on-the-job training and/or simulation)?	☐ Yes	1
		☐ No	0
4	Has the port cleaning staff received basic training on IPC? (If yes state when)	☐ Yes	1
		☐ No	0
5	How frequently do the cleaners receive training regarding IPC in your station?	☐ Never or rarely	0
		☐ New employee orientation	0.2
		☐ New employee orientation and regular (at least annually) IPC training but not mandatory	0.5
		☐ New employee orientation and regular (at least annually) mandatory IPC training	1
6	Has the administrative and managerial staff receive basic training on IPC?	☐ Yes	1
		☐ No	0
	**Subtotal score**	**/6**	

iii. Monitoring of IPC practices

PoE staff IPC practices should be monitored routinely for efficiency

No.	Questions	Answer	Score
1	Are there periodic evaluations or monitoring of IPC compliance? *(Verify that monitoring report is available)*	☐ Yes	1
		☐ No	0
2	If yes, are there trained personnel responsible for the monitoring of IPC practices?	☐ Yes	1
		☐ No	0
3	If yes, is there a well-defined structured checklist to support the monitoring? *(Verify that monitoring checklist is available)*	☐ Yes	1
		☐ No	0
4	If yes, which of the following processes and indicators are monitored?		
i	Hand hygiene compliance	☐ Yes	1
		☐ No	0
ii	Cleaning and disinfection of the environment	☐ Yes	1
		☐ No	0
iii	Waste management	☐ Yes	1
		☐ No	0
5	How frequently do you monitor these indicators?	☐ Never	0
		☐ Periodically, but no regular schedule	0.5
		☐ At least annually	1
6	Do you give feedback reports on the state of the IPC activities/performance? *(Verify that feedback report is available)*	☐ Yes	1
		☐ No	0
7	If yes, who do you share the monitoring report with?		
I	Port health staff *(Verify)*	☐ Yes	1
		☐ No	0
ii	Port health management and senior administration *(Verify)*	☐ Yes	1

		☐ No	0
	**Subtotal score**	**/10**	

iv. Screening station

There should be a well-equipped screening station at the entrance of the PoE with a hand hygiene station (with soap and water and/or hand sanitizer). Personal protective equipment (PPE) should be available in adequate quantities for use when necessary.

No.	Questions	Answer	Score
1	Is there a screening station at the entrance of the PoE? (Verify)	☐ Yes	1
		☐ No	0
2	If yes, is the screening station manned by a port health staff?	☐ Yes	1
		☐ No	0
3	Are the following screening equipment and materials available at the screening station? *(Indicate **YES** only if screening equipment and materials are available)*		
i	Algorithm for screening	☐ Yes	1
		☐ No	0
ii	Screening register/questionnaire	☐ Yes	1
		☐ No	0
iii	Functional Infrared thermometer	☐ Yes	1
		☐ No	0
iv	Functional hand hygiene station (with soap, water, and/or alcohol-based hand rub)?	☐ Yes	1
		☐ No	0
4	Are water services available and of sufficient quantity for uses such as hand hygiene, personal hygiene and drinking) *(Verify, note quantity)*	☐ None	0
		☐ Some	0.2
		☐ Mostly	0.5
		☐ Fully	1
5	Are PPEs (Face mask, face shield) available at the screening station in sufficient quantity? *(Verify)*	☐ None	0
		☐ Yes but not in sufficient quantity	0.5
		☐ Yes, in sufficient quantity	1

	**Subtotal score**	/8	

v. Cleaning and sanitation

Clean and hygienic environments are necessary for both staff and travelers

No.	Questions	Answer	Score
1	Are there dedicated personnel for cleaning at the PoE?	☐ Yes	1
		☐ No	0
2	If yes, how frequently is the cleaning carried out?	☐ Yes	1
		☐ No	0
3	Is there a well-maintained toilet facility?	☐ None	0
		☐ Yes, not well maintained	0.5
		☐ Yes, well maintained	1.0
4	Are appropriate and well-maintained materials for cleaning (for example, detergent, mops, buckets and water) available? *(Observe)*	☐ None	0
		☐ Yes but not in sufficient quantity	0.5
		☐ Yes, in sufficient quantity	1
5	Are waste collection containers available for waste in close proximity to waste generation points? *(Observe)*	☐ Yes	1
		☐ No	0

6	If yes, is a functional burning pit/fenced waste dump or municipal pick-up available for disposal of waste? *(Observe)*	☐ Yes	1
		☐ No	0
	**Subtotal score**	**/6**	

vi. Isolation facility

There should be a designated area/private room(s) if available that has a comfortable temperature and good ventilation assigned for travelers suspected of infectious disease for further assessment

No.	Questions	Answer	Score
1	Is there a designated isolation area for suspected/sick travelers until further evaluation? *(Observe)*	☐ Yes	1
		☐ No	0
2	Is the isolation area in a permanent structure?	☐ Yes	1
		☐ No	0
3	Is the isolation area standard with separate toilet and waste management?	☐ Yes	1
		☐ No	0
4	Are there trained port health staff to identify travelers with suspected priority disease (e.g. COVID-19)?	☐ Yes	1
		☐ No	0
5	If yes, are travelers that meet the case definition for suspected priority disease immediately isolated/separated from the others?	☐ Yes	1

		☐ No	0
6	Are travelers with respiratory symptoms identified on arrival, given a medical mask and guidance on cough etiquette? *(Availability of face mask)*	☐ Yes	1
		☐ No	0
	**Subtotal score**	**/6**	

vii. System of referral

PoEs should have a system for referral of suspected/sick travelers to healthcare facilities for further assessment and treatment

No.	Questions	Answer	Score
1	Is there a system for the referral of symptomatic travelers with suspected priority diseases to healthcare facilities? *(SOP for referrals)*	☐ Yes	1
		☐ No	0
2	If yes, does this PoE have key information regarding the referral health facilities? (Information such as health facility address, the phone number, distance from PoE and map of routes to the health facility)	☐ Yes	1
		☐ No	0
3	Is there a means of transportation for suspected travelers to the identified healthcare facilities? (if yes please state)	☐ Yes	1
		☐ No	0
4	Are there informative posters/IEC materials on COVID-19 at this PoE? E.g. observing hand hygiene, respiratory hygiene, physical distancing, signs & symptoms.	☐ Yes	1

	(confirm *COVID-19 information posters)*	☐ No	0
5	Does this PoE have channels and procedures for communicating health measures on arrival and departure? *(SOP for risk communication/leaflet/posters)*	☐ Yes	1
		☐ No	0
	**Subtotal score**	**/5**	
